# Challenges of Ozone Therapy in Periodontal Regeneration: A Narrative Review and Possible Therapeutic Improvements

**DOI:** 10.3390/cimb47100811

**Published:** 2025-10-01

**Authors:** Nada Tawfig Hashim, Rasha Babiker, Vivek Padmanabhan, Md Sofiqul Islam, Sivan Padma Priya, Nallan C. S. K. Chaitanya, Riham Mohammed, Shahistha Parveen Dasnadi, Ayman Ahmed, Bakri Gobara Gismalla, Muhammed Mustahsen Rahman

**Affiliations:** 1 Department of Periodontics, RAK College of Dental Sciences, RAK Medical & Health Sciences University, Ras-AlKhaimah 12973, United Arab Emirates; 2 Department of Physiology, RAK College of Medical Sciences, RAK Medical & Health Sciences University, Ras-AlKhaimah 11127, United Arab Emirates; 3 Department of Pediatric and Preventive Dentistry, RAK College of Dental Sciences, RAK Medical & Health Sciences University, Ras-AlKhaimah 12973, United Arab Emirates; 4 Department of Operative Dentistry, RAK College of Dental Sciences, RAK Medical & Health Sciences University, Ras-AlKhaimah 12973, United Arab Emirates; sofiqul.islam@rakmhsu.ac.ae; 5 Department of Oral Pathology, RAK College of Dental Sciences, RAK Medical & Health Sciences University, Ras-AlKhaimah 12973, United Arab Emirates; 6 Department of Oral Medicine and Radiology, RAK College of Dental Sciences, RAK Medical & Health Sciences University, Ras-AlKhaimah 12973, United Arab Emirates; 7 Department of Oral Surgery, RAK College of Dental Sciences, RAK Medical & Health Sciences University, Ras-AlKhaimah 12973, United Arab Emirates; 8 Department of Orthodontics, RAK College of Dental Sciences, RAK Medical & Health Sciences University, Ras-AlKhaimah 12973, United Arab Emirates; 9 Department of Periodontology and Implantology, Nile University, Khartoum 11115, Sudan; 10 Department of Oral Rehabilitation, Faculty of Dentistry, University of Khartoum, Khartoum 11115, Sudan

**Keywords:** periodontitis, ozone therapy, oxidative stress, periodontal ligament fibroblasts, extracellular matrix, NF-κB, matrix metalloproteinases, bioactive compounds, nanocarrier drug delivery

## Abstract

Ozone (O_3_) has re-emerged in periodontology for its antimicrobial, oxygenating, and immunomodulatory actions, yet its role in regeneration remains contentious. This narrative review synthesizes current evidence on adjunctive ozone use in periodontal therapy, delineates cellular constraints—especially in periodontal ligament fibroblasts (PDLFs)—and explores mitigation strategies using bioactive compounds and advanced delivery platforms. Two recent meta-analyses indicate that adjunctive ozone with scaling and root planing yields statistically significant reductions in probing depth and gingival inflammation, with no significant effects on bleeding on probing, plaque control, or clinical attachment level; interpretation is limited by heterogeneity of formulations, concentrations, and delivery methods. Mechanistically, ozone imposes a dose-dependent oxidative burden that depletes glutathione and inhibits glutathione peroxidase and superoxide dismutase, precipitating lipid peroxidation, mitochondrial dysfunction, ATP depletion, and PDLF apoptosis. Concurrent activation of NF-κB and upregulation of IL-6/TNF-α, together with matrix metalloproteinase-mediated extracellular matrix degradation and tissue dehydration (notably with gaseous applications), further impairs fibroblast migration, adhesion, and ECM remodeling, constraining regenerative potential. Emerging countermeasures include co-administration of polyphenols (epigallocatechin-3-gallate, resveratrol, curcumin, quercetin), coenzyme Q10, vitamin C, and hyaluronic acid to restore redox balance, stabilize mitochondria, down-modulate inflammatory cascades, and preserve ECM integrity. Nanocarrier-based platforms (nanoemulsions, polymeric nanoparticles, liposomes, hydrogels, bioadhesive films) offer controlled ozone release and co-delivery of protectants, potentially widening the therapeutic window while minimizing cytotoxicity. Overall, current evidence supports ozone as an experimental adjunct rather than a routine regenerative modality. Priority research needs include protocol standardization, dose–response definition, long-term safety, and rigorously powered randomized trials evaluating bioactive-ozone combinations and nanocarrier systems in clinically relevant periodontal endpoints.

## 1. Background

Periodontal disease is one of the most prevalent and debilitating oral health conditions, affecting millions of individuals worldwide [1]. It is a chronic inflammatory condition characterized by progressive destruction of the periodontal ligament, cementum, and alveolar bone [2,3]. According to the Global Burden of Disease Study, severe periodontitis affects more than one billion people globally, ranking among the most widespread chronic diseases [4]. Growing evidence also links periodontitis to systemic disorders such as cardiovascular disease, diabetes mellitus, respiratory conditions, and adverse pregnancy outcomes [1,5,6,7]. These associations are believed to result from the systemic dissemination of oral bacteria and the persistent inflammatory burden, highlighting the importance of periodontitis as both an oral and public health concern [5].

**Figure 1:**
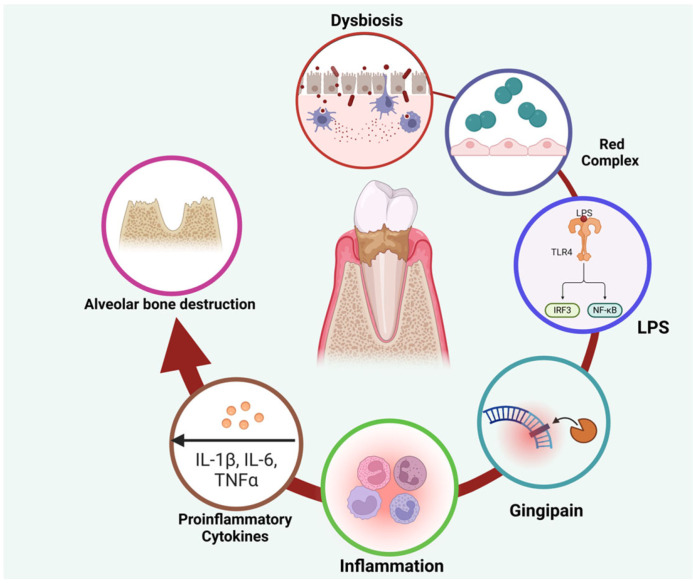
This figure illustrates the pathogenic mechanisms underlying periodontal disease progression. Oral microbial dysbiosis, particularly the overgrowth of the red complex species, promotes the release of virulence factors such as lipopolysaccharides (LPS) and gingipains. These molecules activate host immune receptors, including TLR4, initiating an inflammatory cascade. The subsequent upregulation of proinflammatory cytokines (IL-1β, IL-6, TNF-α) perpetuates tissue inflammation and contributes to alveolar bone destruction, which represents a key hallmark of periodontitis. Created with Biorender.com.

The pathogenesis of periodontitis is primarily driven by dysbiotic microbial communities that initiate an inflammatory cascade involving the upregulation of cytokines such as interleukin-6 (IL-6), tumor necrosis factor-alpha (TNF-α), and nuclear factor kappa B (NF-κB). This cascade activates matrix metalloproteinases (MMPs) and promotes osteoclastic bone resorption, thereby accelerating tissue destruction [8,9,10,11] (Figure 1). In advanced stages, the immune system fails to eliminate the microbial biofilm, leading to chronic inflammation and progressive breakdown of periodontal tissues [11].

**Figure 2:**
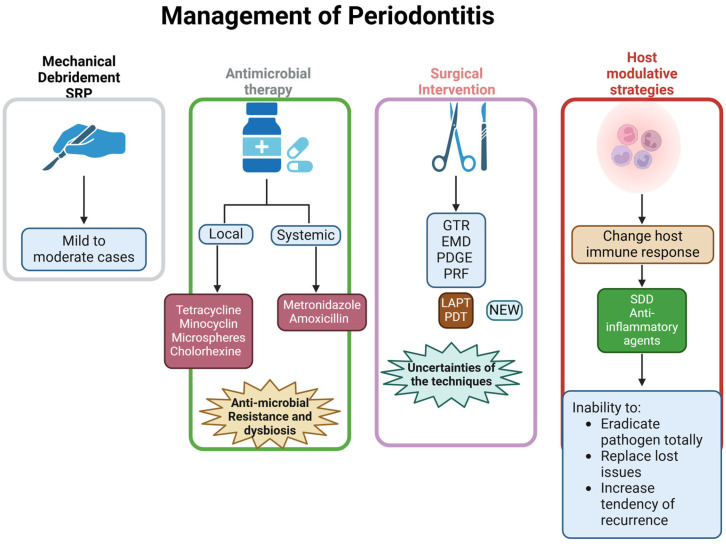
This figure outlines the comprehensive management strategies for periodontitis. Treatment begins with mechanical debridement (scaling and root planing) for mild to moderate cases. Antimicrobial therapy can be administered locally or systemically to target bacterial pathogens. Surgical interventions, including regenerative techniques like GTR, EMD, and PRF, are used in advanced cases. Host modulatory strategies aim to alter the immune response using agents like sub-antimicrobial dose doxycycline (SDD) and anti-inflammatory drugs. Despite these approaches, challenges such as antimicrobial resistance, tissue regeneration, and recurrence remain. Created with Biorender.com.

Current therapeutic strategies of periodontitis include mechanical debridement, antimicrobial agents, surgical procedures, and host modulation [12]. Traditional scaling and root planing (SRP) is the cornerstone of periodontal therapy, and it aims to eliminate all bacterial plaque load [13]. Although SRP is effective in mild and moderate disease, its efficacy diminishes in advanced cases with deep periodontal pockets and severe alveolar bone loss [14]. In such cases, adjunctive antimicrobial approaches, including locally delivered agents such as tetracycline fibers, minocycline microspheres, and chlorhexidine gels, as well as systemic antibiotics such as metronidazole and amoxicillin, have been employed to enhance the therapeutic outcomes [15,16,17]. However, concerns about the growing threat of antibiotic resistance and the negative impacts of disrupting the natural oral microbiome have led to a focus on alternative therapeutic approaches [18]. Regenerative techniques, including guided tissue regeneration (GTR), enamel matrix derivatives (EMD), and growth factors such as platelet-derived growth factor (PDGF) and platelet-rich fibrin (PRF), have been investigated for their potential to restore lost periodontal structures [19,20,21] (Figure 2). These techniques work by creating space for cells to regenerate, promoting cell proliferation and differentiation, and providing bioactive stimuli to stimulate tissue ingrowth [20,21]. Despite encouraging results, clinical success remains unpredictable due to patient-related factors such as smoking, diabetes, and immune status [22]. Technological advances have also introduced laser-assisted periodontal therapy (LAPT) and photodynamic therapy (PDT), which can decontaminate periodontal pockets and promote tissue repair [23]. Host modulation therapies, including subantimicrobial-dose doxycycline (SDD) and novel anti-inflammatory agents, are also under study for their ability to regulate cytokine activity and reduce connective tissue destruction [24,25]. Nonetheless, existing strategies continue to face major challenges, including incomplete biofilm eradication, limited regenerative potential, and high recurrence rates [26]. These limitations have drawn attention to adjunctive therapeutic modalities, among which ozone therapy has emerged as a promising option. Ozone has demonstrated potent antimicrobial activity and potential benefits in redox regulation and tissue healing, yet its clinical application remains controversial because of dose-dependent cytotoxicity [27,28]. The present narrative review aims to assess the role of Ozone therapy in periodontal management, focusing on its antimicrobial and biological effects, outlining the major limitations associated with its clinical use, and evaluating whether bioactive compounds and advanced delivery systems may offer strategies to overcome these drawbacks.

Although this work is presented as a narrative review, we undertook a focused literature search to ensure the inclusion of the most relevant and up-to-date evidence. Electronic databases (PubMed, Scopus, and Web of Science) were searched for studies published between 1987 and 2025 using predefined keywords such as ‘ozone therapy’, ‘periodontitis’, ‘periodontal regeneration’, ‘oxidative stress’, ‘fibroblast viability’, ‘nanoparticles’, and ‘bioactive compounds’. Additional sources were identified by hand-searching the reference lists of key papers. The initial search yielded 356 records, of which duplicates, irrelevant articles, and non-English publications were excluded. A total of 124 articles were screened, and 78 were ultimately selected as most relevant to ozone therapy, its biological effects, and adjunctive strategies in periodontal regeneration. While a formal systematic protocol was not followed, we aimed to minimize bias by using multiple databases, applying clear inclusion and exclusion criteria, and transparently reporting the study selection process to enhance reproducibility.

## 2. Ozone Therapy: A Paradigm Shift in Periodontal Treatment

**Figure 3:**
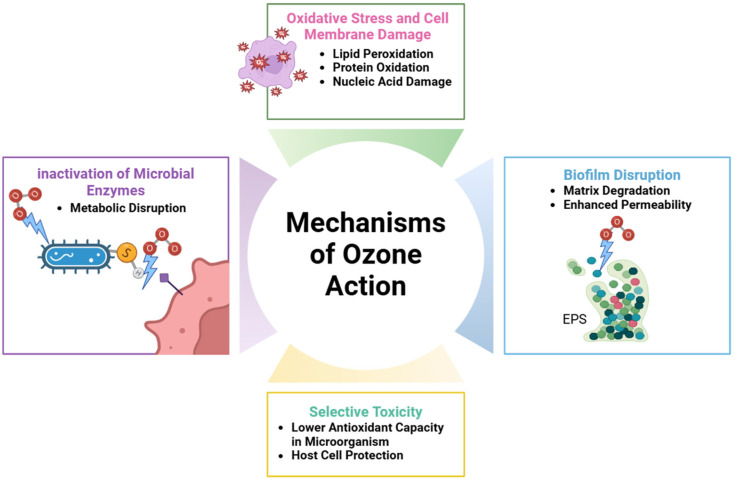
This figure illustrates the various mechanisms through which ozone exerts its antimicrobial effects. Ozone induces oxidative stress leading to lipid peroxidation, protein oxidation, and nucleic acid damage. It disrupts microbial biofilms by degrading the extracellular matrix and increasing permeability. Ozone also inactivates microbial enzymes, causing metabolic disruption. Additionally, its selective toxicity arises from microorganisms’ lower antioxidant capacity, allowing host cells to remain protected. Created with Biorender.com.

**Figure 4:**
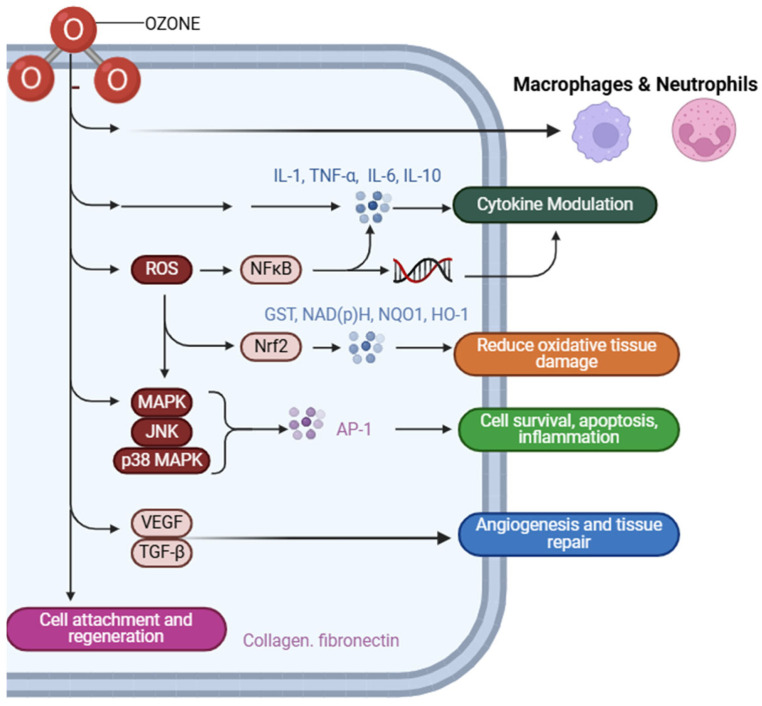
This figure depicts the immunomodulatory effects of ozone on macrophages and neutrophils. Ozone influences the release of key cytokines such as IL-1β, TNF-α, and IL-6, modulating the inflammatory response. It enhances antioxidant defense via activation of Nrf2 and promotes nitric oxide (NO) production. These actions collectively result in oxidative modulation, suppression of excessive inflammation, regulation of matrix metalloproteinases (MMPs), and stimulation of tissue repair mechanisms, including collagen synthesis and cell attachment. Created with Biorender.com.

Ozone therapy has gained notable interest in the periodontal field because of its antimicrobial, anti-inflammatory, and oxygenating properties [27]. Chemically, ozone is a highly reactive triatomic molecule (O_3_) that quickly decomposes into oxygen (O_2_) and reactive oxygen species (ROS). The ROS produced are strong oxidants that can damage microbial cell membranes, proteins, and nucleic acids. This mechanism underpins ozone’s broad-spectrum antimicrobial activity against various pathogens, including bacteria, viruses, fungi, and protozoa [29]. In periodontal treatments, studies have demonstrated that ozone is highly effective against key pathogens involved in disease progression, such as *Porphyromonas gingivalis* (*P. gingivalis)*, *Tannerella forsythia* (*T. forsythia*), and *Aggregatibacter actinomycetemcomitans* (*A. actinomycetemcomitans*) [30] (Figure 3 and Figure 4).

**Figure 5:**
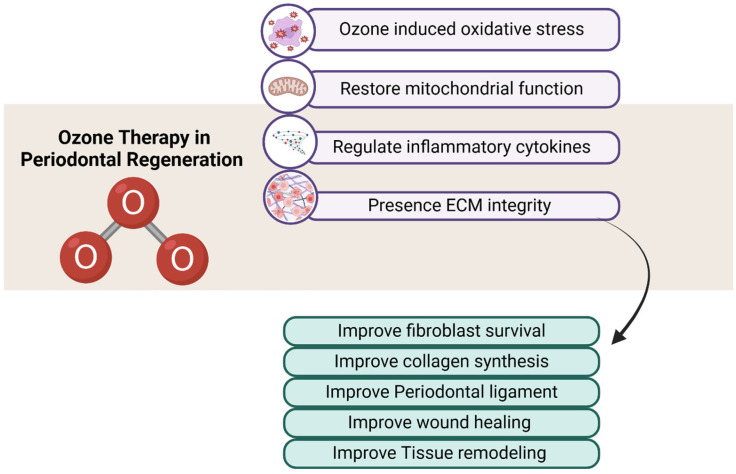
This figure depicts schematic representation of ozone therapy in periodontal regeneration. Ozone acts through multiple cellular mechanisms, including induction of oxidative stress, restoration of mitochondrial function, regulation of inflammatory cytokines, and preservation of extracellular matrix integrity (purple boxes). These mechanisms contribute to enhanced fibroblast survival, increased collagen synthesis, stabilization of the periodontal ligament, improved wound healing, and tissue remodeling (green boxes). The central red O_3_ symbol highlights ozone as the therapeutic agent. Color coding indicates functional grouping: purple = cellular/molecular mechanisms, green = biological/clinical outcomes, red = ozone molecule. Created with Biorender.com.

**Figure 6:**
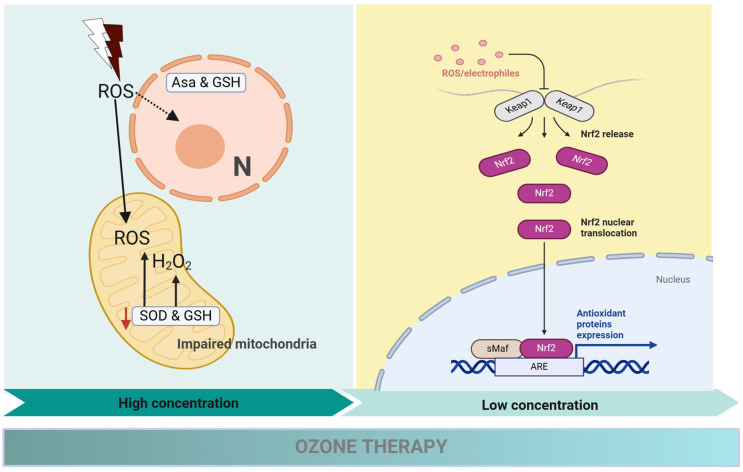
This figure displays the dose-dependent effects of ozone therapy on cellular pathways. At high concentrations (left panel), ozone generates excessive reactive oxygen species (ROS), leading to H_2_O_2_ accumulation, impaired mitochondrial function, and depletion of antioxidants such as ascorbate (Asa), glutathione (GSH), and superoxide dismutase (SOD). This imbalance promotes oxidative stress and cellular injury. At low concentrations (right panel), mild ROS/electrophile signaling activates the Nrf2–Keap1 pathway. Nrf2 is released from Keap1, translocates into the nucleus, and binds to the antioxidant response element (ARE), driving transcription of cytoprotective proteins and enhancing antioxidant defense. Black solid arrows = progression of signaling events or molecular processes. Red downward arrow = inhibition or depletion of antioxidant defenses. Dashed arrow = nuclear translocation of Nrf2. Created with Biorender.com.

The primary benefit of Ozone therapy is its remarkable ability to significantly improve local oxygen levels in periodontal tissues, as well as its capacity to effectively stimulate fibroblast proliferation, encourage angiogenesis, and support overall wound healing [31] (Figure 5). When applied at low concentrations, Ozone has been shown to activate the Nrf2–Keap1 signaling pathway. This pathway is a key regulator of cellular responses to oxidative stress, leading to increased expression of antioxidant response elements (AREs) [28]. This adaptive process not only enhances cellular resistance to oxidative damage but also promotes tissue repair and bolsters immune functions [28] (Figure 6). However, it is important to note that at concentrations exceeding the therapeutic range, Ozone can cause excessive production of reactive oxygen species, resulting in oxidative stress, apoptosis, mitochondrial damage, and impaired fibroblast migration [32]. Cellular damage also arises from the intracellular depletion of glutathione (GSH) and the inactivation of vital antioxidant enzymes such as glutathione peroxidase and superoxide dismutase (SOD) [33] (Figure 6). Another significant drawback of Ozone excess is the activation of inflammatory signaling pathways, with elevated levels of proinflammatory cytokines like IL-6 and TNF-α, which promote tissue destruction rather than regeneration [34]. Additionally, ozone has been linked to increased activity of matrix metalloproteinases (MMPs), leading to the degradation of collagen and other components of the extracellular matrix (ECM), thereby causing further damage to periodontal tissues [35].

## 3. Challenges of Ozone Therapy in Periodontal Fibroblasts

O_3_ therapy, owing to its antimicrobial potential and ability to disrupt biofilms, has been regarded as a promising adjunctive approach in periodontal treatment. Its capacity to enhance conventional therapy has generated considerable interest. Nonetheless, recent systematic reviews and meta-analyses have offered a more cautious interpretation of its role in periodontal care. Liu et al. (2025) demonstrated that while ozone therapy combined with scaling and root planing (SRP) resulted in statistically significant reductions in probing depth and gingival inflammation, it did not produce significant improvements in bleeding on probing, plaque control, or clinical attachment gain [36]. Similarly, Pardo et al. (2025) reported statistically significant improvements in probing pocket depth and gingival indices, with moderate certainty of evidence, yet underscored that these benefits were clinically limited and highly heterogeneous due to inconsistencies in ozone formulations, concentrations, and delivery techniques [37]. Collectively, these findings suggest that although ozone exhibits antimicrobial and anti-inflammatory properties in vitro and in animal studies, translation into reproducible clinical outcomes remains weak. Both meta-analyses highlighted methodological shortcomings, including the absence of standardized treatment protocols, limited follow-up periods, and the small scale of available clinical trials. Consequently, current evidence does not support ozone therapy as a reliable alternative or superior adjunct to established periodontal therapies.

**Figure 7:**
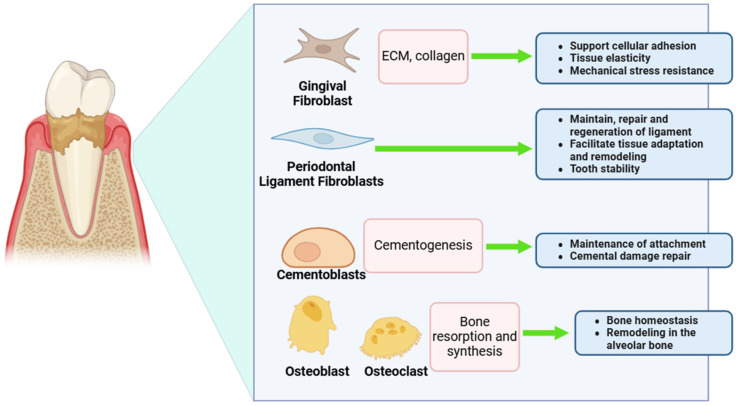
This figure highlights key periodontal cell lines used in periodontal disease research and their specific roles. Gingival fibroblasts contribute to ECM and collagen production, supporting cell adhesion and mechanical resilience. Periodontal ligament fibroblasts are vital for maintaining and remodeling the ligament and ensuring tooth stability. Cementoblasts regulate cementogenesis, aiding in attachment and repair, while osteoblasts and osteoclasts coordinate bone resorption and synthesis, essential for alveolar bone homeostasis and remodeling. Created with Biorender.com.

Beyond clinical outcomes, ozone therapy raises further complexities regarding its effects on periodontal ligament fibroblasts (PDLFs) [38,39]. Several mechanistic challenges limit its regenerative potential, particularly in relation to fibroblast function, extracellular matrix (ECM) remodeling, and inflammation control (Figure 7). Ozone-induced oxidative stress represents a major obstacle, leading to rapid depletion of glutathione (GSH), a key intracellular antioxidant responsible for scavenging reactive oxygen species (ROS) [39]. This depletion, coupled with the inactivation of glutathione peroxidase and superoxide dismutase (SOD), accelerates lipid peroxidation, disrupts mitochondrial function, and promotes fibroblast apoptosis [40,41]. Loss of mitochondrial integrity further diminishes ATP production, thereby impairing fibroblast migration and reducing ECM remodeling capacity—processes indispensable for periodontal regeneration [42]. In parallel, ozone exposure induces a heightened inflammatory response, elevating the production of IL-6 and TNF-α and activating nuclear factor-κB (NF-κB) [28]. While transient inflammation is necessary for repair, prolonged or excessive inflammatory activity adversely affects fibroblast viability, delays healing, and can ultimately drive tissue degradation rather than regeneration [43]. The impact on ECM stability is another critical concern, as ozone activates matrix metalloproteinases (MMPs), which degrade collagen and other ECM components, thereby undermining periodontal structural integrity [44]. Supporting this, a study investigating ozone oil therapy in diabetic patients revealed that although microbial load was significantly reduced, fibroblast viability and migration were not significantly improved, underscoring that ozone alone is insufficient for periodontal regeneration [45]. Additional adverse effects have also been reported, including oxidative stress, excessive inflammation, localized tissue dehydration, and diminished fibroblast adhesion [46]. These limitations are particularly pronounced with gaseous ozone, as exposure can desiccate fibroblast surfaces, restricting their ability to adhere, migrate, and proliferate within the ECM. Consequently, the regenerative potential of ozone therapy is constrained, as inadequate ECM availability further limits the capacity of fibroblasts to contribute to periodontal healing [46].

Recent findings on Ozone ultrafine bubble water (OUFBW) have provided deeper insights into the cellular response of periodontal ligament fibroblasts (PDLFs) to oxidative stress [43]. Exposure to OUFBW leads to the generation of ROS, triggering oxidative stress-adaptive pathways such as the Nrf2-Keap1 system. While this response may enhance cellular antioxidant defenses, prolonged activation can lead to mitochondrial depolarization, ATP depletion, and fibroblast apoptosis, limiting the potential for tissue regeneration. Additionally, the activation of mitogen-activated protein kinase (MAPK) signaling, particularly p38 MAPK, further exacerbates inflammation, leading to the upregulation of pro-inflammatory cytokines such as IL-6 and TNF-α. Given these findings, it is evident that while Ozone therapy exerts antimicrobial effects and influences fibroblast activity, its oxidative burden requires modulation through bioactive compounds. Epigallocatechin-3-gallate (EGCG), resveratrol, curcumin, and quercetin have demonstrated the ability to counteract ozone-induced oxidative stress, stabilize mitochondrial function, and regulate inflammatory cytokine expression. Moreover, hyaluronic acid (HA) plays a crucial role in protecting fibroblasts from dehydration and oxidative damage, ensuring ECM integrity post-ozone exposure [43].

## 4. Counteracting Ozone’s Limitations with Bioactive Compounds

**Figure 8:**
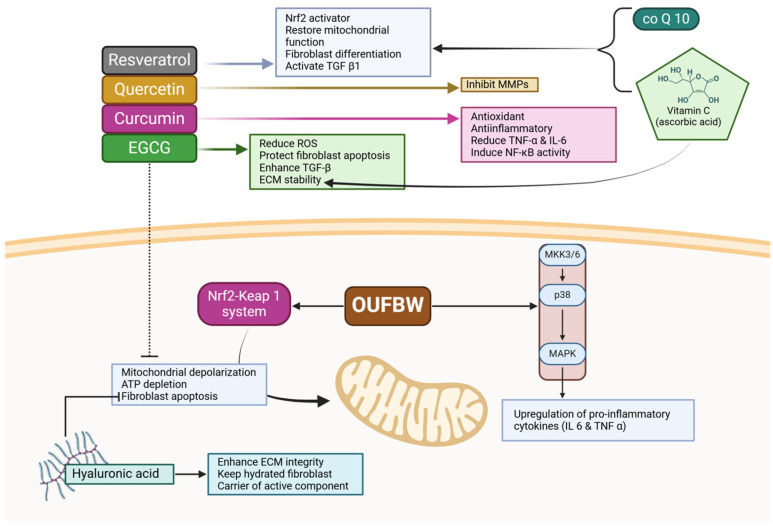
This figure illustrates the protective effects of natural compounds and antioxidants on fibroblast function and mitochondrial integrity in periodontal therapy. Polyphenols like resveratrol, quercetin, curcumin, and EGCG activate the Nrf2 pathway, enhance mitochondrial function, and reduce oxidative stress. Coenzyme Q10 and vitamins C and E support antioxidant defenses and inhibit MAPKs. Hyaluronic acid preserves ECM integrity and hydration. Together, these agents modulate inflammatory signaling pathways, reduce pro-inflammatory cytokines, and help maintain fibroblast viability and tissue regeneration. Created with Biorender.com.

Ozone therapy is associated with various challenges [47]. Therefore, bioactive compounds with antioxidant, anti-inflammatory, and ECM-stabilizing properties could be utilized to mitigate these effects while preserving the therapeutic benefits of ozone [48]. Several natural bioactive agents have been identified as potential modifiers of ozone therapy to help maintain the regenerative capacity of fibroblasts when exposed to ozone. EGCG, a polyphenol derived from green tea, has exhibited high efficacy in scavenging Ozone-induced ROS and protecting against fibroblast apoptosis, but only when the apoptosis is induced by oxidative stress [49,50]. Concurrently, it enhances TGF-β production, essential for ECM remodeling and collagen production [50] (Figure 8). The strong antioxidant activity of EGCG alleviates the consequences of glutathione depletion, thereby restoring the redox homeostasis of fibroblasts and consequently ensuring ECM stability [49]. Similarlyy, resveratrol, a known Nrf 2 activator, restores mitochondrial function and promotes fibroblast differentiation. Resveratrol operates through the same mechanism as Ozone therapy; it activates TGF-β 1 and oxidative stress pathways to reduce the antimicrobial effects of ozone therapy [49]. Recent studies highlight resveratrol’s capacity to enhance antioxidant defenses, reduce mitochondrial dysfunction, and lessen oxidative damage, emphasizing its therapeutic potential for redox-mediated tissue injury [50]. Thus, resveratrol is a promising bioactive molecule that may enhance fibroblast viability in ozone-treated periodontal tissues. Beyond antioxidants, curcumin displays significant anti-inflammatory activity and reduces the production of TNF-α and IL-6, preventing excessive ozone-induced inflammation [49]. Curcumin influences NF-κB activity, assisting in the regulation of the immune response to ensure that ozone-induced inflammation does not impede the healing of periodontal tissues. In addition to curcumin, quercetin offers added matrix protection by inhibiting MMPs, thus shielding collagen from degradation and enhancing the strength of the periodontium [49,51]. Moreover, HA is employed to prevent fibroblast dehydration and maintain ECM integrity. Considering that ozone therapy can induce tissue dehydration in the surrounding area, HA helps to keep fibroblasts fully hydrated, active, and capable of optimal migration and adhesion within the periodontal niche. Furthermore, HA serves as a carrier for other active components, enhancing the shelf life and potency of these components when used alongside ozone therapy [52]. Mitochondrial dysfunction and ATP depletion following ozone exposure also require intervention; Coenzyme Q 10 (CoQ 10) and Vitamin C are highly effective in this regard [53]. Therefore, CoQ 10 regulates mitochondrial activity, enabling fibroblasts to sustain their energy homeostasis, while Vitamin C is crucial for collagen formation and ECM regulation, both vital for the healing of periodontal tissues [54] (Figure 8).

Taken together, the mixed clinical outcomes with ozone therapy appear to reflect limitations in delivery and dosing rather than an absence of biological efficacy. Its clinical reliability will likely depend on integrated strategies such as co-administration of antioxidants (e.g., EGCG, resveratrol, curcumin, CoQ10, hyaluronic acid) to counteract oxidative burden, combined with advanced delivery systems (nanoparticles, hydrogels, bioadhesive films) designed to sustain local availability and improve periodontal pocket retention. It is important to emphasize that most data on these adjunctive bioactive compounds derive from preclinical models. Among them, hyaluronic acid and CoQ10 have advanced into early clinical studies in periodontal patients, with small-scale trials suggesting benefits in wound healing and attenuation of oxidative stress. However, robust randomized clinical trials investigating polyphenols, resveratrol derivatives, or other novel antioxidants in human periodontitis remain largely absent, underscoring the gap between promising laboratory findings and evidence-based clinical application.

## 5. Toward a Bioactive-Ozone Therapeutic Model

To maximize Ozone therapy’s antimicrobial potential while counteracting its negative effects on fibroblasts and ECM remodeling, a multi-step bioactive-Ozone therapeutic model can be adopted. This involves preconditioning fibroblasts with antioxidant agents such as NAC and EGCG before ozone exposure, followed by controlled Ozone application for microbial elimination, and finally, post-treatment supplementation with CoQ10, Vitamin C, Curcumin, and HA to support fibroblast viability, inflammation control, and ECM remodeling. This strategy ensures that Ozone therapy remains a precise and effective tool for periodontal regeneration rather than an oxidative burden on host tissues.

## 6. A Novel Bioactive-Ozone Nanocarrier System for Enhanced Periodontal Regeneration

A significant drawback of Ozone treatment is its short-lived effectiveness due to rapid decomposition into other reactive oxygen species (ROS) and its potential to cause cell damage and death at high concentrations by overwhelming the body’s antioxidant defenses and inducing harmful oxidative stress [28]. An Ozone-loaded nanocarrier system is proposed as a novel approach to achieve this goal, which would enable the targeted and extended administration of Ozone and other beneficial compounds (EGCG, resveratrol, curcumin, CoQ10, and hyaluronic acid) [55]. Nanotechnology-based drug delivery systems, including nanoemulsions, polymeric nanoparticles, liposomes, and hydrogels, are employed to enhance the controlled and targeted delivery of therapeutic agents for periodontitis treatment and to promote tissue regeneration while mitigating oxidative stress induced by ozone therapy [56,57]. This is due to the fact that traditional Ozone administration produces a rapid increase of ROS that are quickly diminished, while this approach enables a continuous, low-dose release of ozone for antibacterial effects with little cytotoxicity to fibroblasts [58]. This hybrid system has several benefits compared to traditional ozone treatment. The regulated administration of ozone reduces oxidative stress by limiting ROS exposure while preserving its bactericidal efficacy [59]. Moreover, the co-encapsulation of antioxidants such EGCG, resveratrol, and CoQ10 mitigates fibroblast toxicity and promotes ECM remodeling by safeguarding fibroblasts from ozone-induced glutathione depletion and mitochondrial dysfunction [60]. The inclusion of HA improves fibroblast adhesion, hydration, and collagen synthesis to mitigate the drying impact of Ozone gas [61]. Moreover, the nanocarriers infused with CoQ10 and Vitamin C augment mitochondrial ATP synthesis and optimize the energy metabolism of fibroblasts to facilitate efficient tissue repair [62]. This nanocarrier-based Ozone delivery technology offers biological advantages together with a less invasive and targeted therapy approach. It may be delivered as an injectable hydrogel, bioadhesive periodontal film, or nanoparticle-infused mouthwash, therefore facilitating its use in clinical practice for both non-surgical and surgical periodontal treatment [63,64]. Furthermore, in instances with significant periodontal problems, this bioactive nanocarrier system may be used in guided tissue regeneration (GTR) membranes or biodegradable scaffolds to facilitate long-term tissue stability and bone regeneration in periodontally compromised areas [65,66]. This method represents a groundbreaking advancement in periodontal therapy, using Ozone therapy, bioactive agents, and nanotechnology to provide novel regeneration procedures [27,67]. This innovative technology may improve periodontal healing and therapy outcomes by addressing issues related to transient Ozone effects, oxidative stress, and unpredictable fibroblast responses. Future research should focus on formulation optimization, in vivo biocompatibility, and long-term efficacy studies to establish this hybrid system as a next-generation periodontal therapy.

## 7. Conclusions

Ozone therapy has demonstrated promising adjunctive benefits in periodontal care through its antimicrobial, oxygenating, and immunomodulatory properties. When combined with conventional approaches such as scaling and root planing, it may enhance periodontal healing and improve treatment outcomes. Nonetheless, its therapeutic success depends on carefully balancing antimicrobial efficacy with the risk of oxidative stress and fibroblast cytotoxicity. Advances in bioactive molecules and nanotechnology-based delivery systems could help mitigate side effects and improve ozone’s regenerative potential. However, its clinical applicability remains constrained by delivery limitations, lack of standardized protocols, and insufficient long-term safety data. Future progress will require rigorously designed randomized clinical trials to establish optimal concentrations, delivery methods, and durability of outcomes. Until such evidence is available, ozone therapy should be regarded as an experimental adjunct rather than a routine component of periodontal treatment.

- Limitations and Future Directions

A major limitation in current evidence is the absence of standardized clinical protocols. Reported concentrations, exposure times, and delivery modes (gaseous, aqueous, oil-based, or nanocarrier systems) vary widely, preventing cross-study comparability and guideline development. In addition, long-term safety data are scarce, with most clinical trials limited to short-term follow-up. While some clinical investigations are ongoing into ozone-based gels and nanocarrier systems, no large multicenter randomized trials have yet established definitive safety and efficacy profiles. These gaps must be addressed before ozone therapy can be integrated into routine periodontal protocols.

It is also unclear how O_3_ interacts with systemic conditions such as diabetes, cardiovascular disease, and immunocompromised states. Considering the bidirectional link between periodontal health and these systemic diseases, future research must elucidate how O_3_ therapy affects outcomes in these high-risk groups.

Finally, more focus should be placed on creating advanced delivery systems that enable controlled O_3_ release with minimal cytotoxicity. Although nanocarriers and bioactive-loaded systems have been suggested, their clinical effectiveness, biocompatibility, and cost-efficiency need thorough evaluation before widespread use.

## Data Availability

No new data were created or analyzed in this study. Data sharing is not applicable to this article.

## Abbreviations

The following abbreviations are used in this manuscript:

ARE	Antioxidant Response Element
ATP	Adenosine Triphosphate
ECM	Extracellular Matrix
EGCG	Epigallocatechin-3-Gallate
EMD	Enamel Matrix Derivative
GSH	Glutathione
GTR	Guided Tissue Regeneration
HA	Hyaluronic Acid
IL	Interleukin
LAPT	Laser-Assisted Periodontal Therapy
MMPs	Matrix Metalloproteinases
NF-κB	Nuclear Factor Kappa B
PRF	Platelet-Rich Fibrin
ROS	Reactive Oxygen Species
SRP	Scaling and Root Planing
SOD	Superoxide Dismutase
TGF-β	Transforming Growth Factor Beta
TNF-α	Tumor Necrosis Factor Alpha
